# Genomic survey, expression profile and co-expression network analysis of *OsWD40 *family in rice

**DOI:** 10.1186/1471-2164-13-100

**Published:** 2012-03-20

**Authors:** Yidan Ouyang, Xiaolong Huang, Zhanhua Lu, Jialing Yao

**Affiliations:** 1 National Key Laboratory of Crop Genetic Improvement and National Center of Plant Gene Research (Wuhan), Huazhong Agricultural University, Wuhan 430070, China; 2 College of Life Science and Technology, Huazhong Agricultural University, Wuhan 430070, China; 3 College of Horticulture and Forestry Science, Huazhong Agricultural University, Wuhan 430070, China

**Keywords:** *Oryza sativa*, Expression profiles, Microarray, *WD40 *gene, Co-expression network

## Abstract

**Background:**

WD40 proteins represent a large family in eukaryotes, which have been involved in a broad spectrum of crucial functions. Systematic characterization and co-expression analysis of *OsWD40 *genes enable us to understand the networks of the WD40 proteins and their biological processes and gene functions in rice.

**Results:**

In this study, we identify and analyze 200 potential *OsWD40 *genes in rice, describing their gene structures, genome localizations, and evolutionary relationship of each member. Expression profiles covering the whole life cycle in rice has revealed that transcripts of *OsWD40 *were accumulated differentially during vegetative and reproductive development and preferentially up or down-regulated in different tissues. Under phytohormone treatments, 25 *OsWD40 *genes were differentially expressed with treatments of one or more of the phytohormone NAA, KT, or GA3 in rice seedlings. We also used a combined analysis of expression correlation and Gene Ontology annotation to infer the biological role of the *OsWD40 *genes in rice. The results suggested that *OsWD40 *genes may perform their diverse functions by complex network, thus were predictive for understanding their biological pathways. The analysis also revealed that *OsWD40 *genes might interact with each other to take part in metabolic pathways, suggesting a more complex feedback network.

**Conclusions:**

All of these analyses suggest that the functions of *OsWD40 *genes are diversified, which provide useful references for selecting candidate genes for further functional studies.

## Background

Proteins characterized by conserved motifs may belong to a gene family, which were represented by structural or functional similarity and evolutionary relationships. WD40 proteins are a group of proteins that are highly conserved in evolution and are extremely abundant across a wide range of eukaryotic organisms [1]. Structurally, these proteins are characterized by the presence of approximately 40 amino acids core region, which contains a glycine-histidine (GH) dipeptide at the N terminus and a tryptophan-aspartate (WD) dipeptide at the C terminus separated by a region of variable lengths [2]. Usually, the WD40 protein contains several tandemly repeated units of such motif, which are required to form the secondary structure [3]. The structure of several WD40 proteins has been determined, suggesting that the WD40 domain folds into a secondary structural of beta propeller despite large levels of sequence diversity. For example, the mammalian Gβ subunit of heterotrimeric GTPases involved in signal transduction forms a beta propeller structure containing seven WD40 repeats [2].

*WD40 *gene family shows low level of sequence conservation with functional diversity in diverse pathways, and many WD40 proteins possess additional domains with other functional activities. Biochemical and structural studies have recognized WD40 proteins to be a broader spectrum of components in cytoplasm and nucleoplasm. They participate in important cellular pathways, including signal transduction, RNA processing, cytoskeleton dynamics, vesicular trafficking, nuclear export, regulation of cell division, and are especially prevalent in chromatin modification and transcriptional regulation [2,4,5]. In the model plant *Arabidopsis thaliana*, the WD 40 proteins have been identified and analyzed comprehensively [5]. The results suggested that these proteins played key roles in plant-specific processes, with diversity in function conferred at least in part by divergence in upstream signaling pathways, downstream regulatory targets and/or structure outside of the WD 40 regions [5].

A common characteristic feature of the WD40 proteins is that, the WD40 domain mediates diverse protein-protein or protein-DNA interactions, thus interplays with multiple proteins to form dynamic complexes and functions as scaffolding protein [4,6]. WD40 domain can mediate molecular recognitions with diverse partners through different sides of its surface. The same WD40 proteins can either recruit different substrates in a similar mode or in distinct ways [6]. Considering the multiple interaction modes and the complex roles in cellular processes, it is difficult to identify the partners and pathways in relationship with WD40 proteins. The availability of high-throughput interactomes from different species enables us to understand the networks of the WD40 proteins more comprehensively [7-10]. For instance, WD40 domains were found to take part in more interaction pairs than any other domain in yeast, and being as the one of the most interacting domains in human interactome datasets [7-10]. Meanwhile, WD40 proteins can also act as a component of protein complexes involved in a variety of pathways [11-14], or offer binding sites for other proteins [15,16], demonstrating that they can interact with the appropriate partner in different processes. The expression profiles in genome-wide scale provide essential data for building the co-expression network, thus allowed us to identify biological processes and gene functions [17]. The comprehensive expression data in CREP database encompassing the entire life cycle of rice (*Oryza sativa*) provided rich information for associating the *WD40 *genes in different pathways by co-expression analysis [18]. And deeper understanding of their structures, expression profiles, interactions and functional diversity will be essential for our study in the detailed cellular processes mediated by WD40 proteins.

Rice is one of the major staple foods for world population. It is also an ideal model species for functional genomic analysis and represents an evolutionary lineage within the monocotyledons. In this study, we discuss three important questions through the genome-wide scan and systematic characterization of *OsWD40 *gene family during the whole life cycle in rice. First, how many members belong to this family and what about their localizations, gene structures and other characteristics? Second, what are the expression patterns of the *OsWD40 *genes, and what is the connection between the expression levels and their gene functions? Third, how does this gene family evolve or what are the evolutionary relationships between these *OsWD40 *genes? Therefore, the answers would provide a solid base for future functional genomic studies of the *OsWD40 *genes in rice.

## Results

### Collection and identification of the *OsWD40 *genes in rice

In order to identify the *OsWD40 *genes in the rice, the consensus protein sequence which is characteristic of *WD40 *genes in eukaryotes, GECKXVLXGHTSTVTCVAFSPDGPLLASGSRDGTIKIWD, was generated by hmmemit from HMM profile (PF00400). We carried out BLASTP analysis using this sequence as a query in MSU database http://rice.plantbiology.msu.edu/index.shtml, with a threshold E value of ≤ 10. A total of 342 sequences were identified as putative *OsWD40 *genes. By removal of different transcripts of the same gene, we identified 234 putative *OsWD40 *genes. These candidates were examined by SMART and Pfam searching for the presence of WD40 domain. Thus, 159 genes with the presence of WD40 domain were confirmed by SMART, and additional 41 genes were identified as containing such domain in Pfam. Therefore, there were a total of 200 *OsWD40 *genes in the rice genome. For convenience, the 200 *OsWD40 *genes were named from *OsWD40-1 *to *OsWD40-200 *according to their positions on pseudomolecules. As the table containing the accession numbers of each *OsWD40 *gene is too large within one printed page, these data were exhibited as the additional file (Additional file 1: Table S1). The detailed information of *OsWD40 *genes were also listed in Additional file 1: Table S1.

Except for the presence of a conserved WD40 domain, the *OsWD40 *genes vary substantially in the size and sequences of their encoded proteins, and their physicochemical properties (Additional file 1: Table S1). The position of the WD40 domain within the protein also varies. The length of OsWD40 proteins varied from 91 to 3787 amino acids. EXPASY analysis suggested that the OsWD40 protein sequences had large variations in isoelectric point (pI) values (ranging from 4.0839 to 10.3354) and molecular weight (ranging from 9.997 kDa to 420.65 kDa) (Additional file 1: Table S1). Only 61 of the 200 *OsWD40 *genes were predicted to be stable proteins, while the rest were unstable. Details on other parameters of protein sequences were shown in Additional file 1: Table S1.

### Classification and phylogenetic analysis of OsWD40 proteins

**Figure 1:**
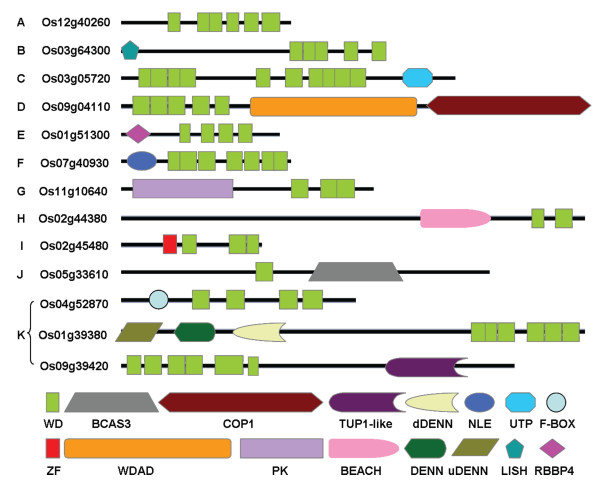
**Structure of representative OsWD40 proteins from each subfamily**. The protein structure is based on the presence of WD40 and other additional domains as identified by SMART. Subfamily name of each corresponding protein belonged to and MSU locus ID are given on the left. Domain abbreviations are: WD, WD40 repeats domain; BCAS3, breast carcinoma amplified sequence 3; COP1, coatomer (COPI) alpha subunit C-terminus; TUP1-like, TUP1-like enhancer of split; dDENN, this region is always found associated with PF02141(DENN); NLE, NLE domain located N-terminal to WD40 repeats; UTP, WD40 associated putative domain; F-box, the F-box domain has a role in mediating protein-protein interactions; ZF, Zinc finger; WDAD, coatomer WD associated region; PK, Protein kinase domain; BEACH, the BEACH domain is usually followed by a series of WD repeats; DENN domain, DENN is a domain involved in Rab- mediated processes or regulation of MAPK signalling pathways; uDENN, this domain is always found associated with DENN; LISH, LisH domain mediates protein dimerisation and tetramerisation; RBBP4, Histone-binding protein RBBP4 or subunit C of CAF1 complex. The length and order of domains represent actual situation in each protein.

The 200 OsWD40 proteins were classified into 11 subfamilies according to their domain compositions (Figure 1). One hundred and forty-five members merely with WD40 domain belonged to subfamily A. Besides WD40 domain, OsWD40 proteins containing several other known functional domains were classified into the following subfamilies. Seven members containing the LisH domain were identified as subfamily B; Five members containing the Utp12, Utp13, Utp15, and Utp21 domain were identified as subfamily C; Four members with the Coatomer WD associated region(WDAD)and/or Coatomer (COPI) alpha subunit C-terminus were identified as D subfamily; E subfamily (4 members) had histone-binding protein RBBP4 or subunit C of CAF1 complex domains before WD40 repeats; F subfamily (3 members) contained NLE (NUC135) domain N terminal to WD40 repeats; G subfamily (4 members) had protein kinase domain or HEAT repeat; H subfamily (3 members) contained the Beige/BEACH domain; I subfamily (2 members) had zinc finger domain; J subfamily (2 members) contained breast carcinoma amplified sequence 3 (BCAS3); K subfamily (21 members) contained other domains including F-BOX, U-BOX and domains with unknown function (Figure 1).

To explore the evolutionary relationships of the *WD40 *genes in rice and *Arabidopsis*, an unrooted phylogenetic tree was generated from alignments of their full-length protein sequences. The phylogenetic analysis revealed that all WD40s were clustered into five distinct groups (Cluster I to Cluster V), comprising 151, 26, 66, 68, and 122 proteins, respectively (Additional file 2: Figure S2). WD40 proteins from rice and *Arabidopsis *are present in all groups. The OsWD40 members were more closely to those in the same clade in *Arabidopsis *than to other OsWD40 proteins in the same species (Additional file 3: Table S3), which indicated synteny and conservation between rice and *Arabidopsis *proteins. Most members in the same groups or subgroups shared one or more domains outside the WD40 domain, thus was consistent with the subfamily definition revealed above.

### Expression profiling of *OsWD40 *genes during the whole life cycle of rice

To study the transcript accumulation of *OsWD40 *genes in the entire life cycle of rice, the expression profiling covering 24 developmental stages (Additional file 4: Table S4) in Minghui 63 were analyzed by Affymetrix rice microarray data in CREP database [18]. Probes for 184 of the 200 *OsWD40 *genes could be identified in the Affymetrix microarray. Thirty-six genes had two probe sets and the higher signal value of the probe sets was used for analysis. Two pairs of genes, *OsWD40-28 *and *OsWD40-42*, as well as *OsWD40-182 *and *OsWD40-193*, shared the same probe sets, respectively. Only 182 genes showed a "present" detection call at p value of 0.05 in at least one of the investigated tissues, whereas two low expressing genes (*OsWD40-151 *and *OsWD40-127*) were either "absent" or "marginal" under these developmental stages. The transcripts of these two genes were at almost undetectable levels in all the stages analyzed, but it is possible that these genes might respond to specific stimuli or their expressions might be limited to specialized cell types that have not been analyzed in this investigation.

**Figure 2:**
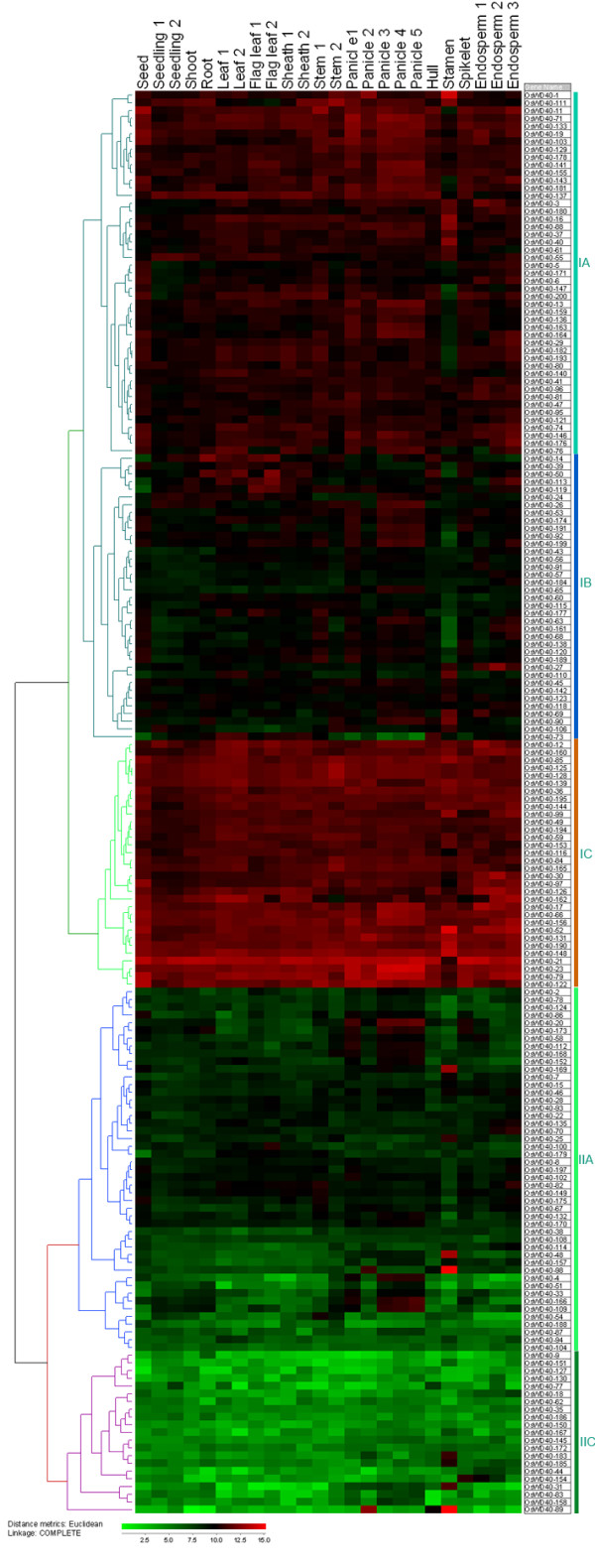
**Expression patterns of *OsWD40 *genes during the life cycle of the rice plant**. Hierarchical cluster display the expression profile for 184 *OsWD40 *genes with probes in the Affymetrix microarray. (Color bar at the base represents log2 expression values: green, representing low expression; black, medium expression; red, high expression). The detailed information of the samples is listed in Additional files 4: Table S4.

A hierarchical cluster displaying the logarithm of average signal values for the 184 *OsWD40 *genes were generated. Based on which the expression patterns of *OsWD40 *genes could be classified into two major groups (Figure 2). One hundred and nineteen genes belonged to Group I, most of which (79%) showed high transcript accumulations (average expression signal higher than 1000) in all the tissues analyzed (Figure 2). These *OsWD40 *genes might play roles in housekeeping functions and gene *OsWD40-21 *had the highest average expression level in the entire life cycle. These 119 genes could be further divided into three subgroups. Subgroup IA consisted of 50 genes, with the average expression signal from 579.5 to 3132.9 (Figure 2). Subgroup IB of 37 genes showed relatively high expression level in a serious of reproductive tissues or vegetative tissues (Figure 2). Notably, the expression levels of 32 genes in subgroup 1C were extremely high, with the average expression signal from 2255 to 10856.5 (Figure 2). Group II consisted of 65 genes, which showed relatively low expression signals or preferential expressions in some tissues (Figure 2). This group can also divide into two subgroups. Subgroup IIA contained 42 genes showing tissue-specific/preferential expressions, e.g., *OsWD40-25*, *48*, *98*, 169 with predominant expression in stamen, and *OsWD40-70 *and *135 *as endosperm preferential expression genes. Interestingly, the expression of eight genes (*OsWD40-4*, *20*, *33*, *51*, *109*, *152*, *166 *and *173*) which was high in panicles decreased in panicle 2 and then increased gradually as the panicles matured. These genes might play essential roles in panicle developing. Subgroup IIB comprised 23 genes, and genes in this subgroup showed almost negligible expressions (Figure 2).

**Figure 3:**
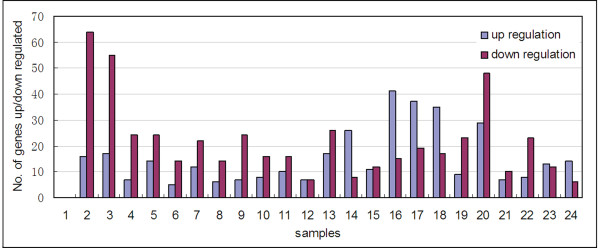
**Differential expressions of *OsWD40 *genes in different stages in Minghui 63 based on microarray analysis**. The detailed information of the samples is listed in Additional files 4: Table S4.

In order to reveal more information in *OsWD40 *expression pattern, genes that showed differential expression during various stages of development in comparison to seed were analyzed. Detailed p values and fold change values were given in Additional file 5: Table S5. Genes considered as preferential expression in a given stage showed tremendous differences (Figure 3, Additional file 5: Table S5). Up-regulated genes mainly accumulated in panicles and stamen, suggesting that *OsWD40 *genes participate in various molecular pathways in flowering development. Surprisingly, although down-regulated genes accumulated in seedlings, they were activated in stamen, either (Figure 3, Additional file 5: Table S5). This result suggested that *OsWD40 *genes might control stamen regulation. They might also play important roles in early developmental stages in seedlings.

### Responses of *OsWD40 *genes under NAA, KT, and GA3 treatments

**Figure 4:**
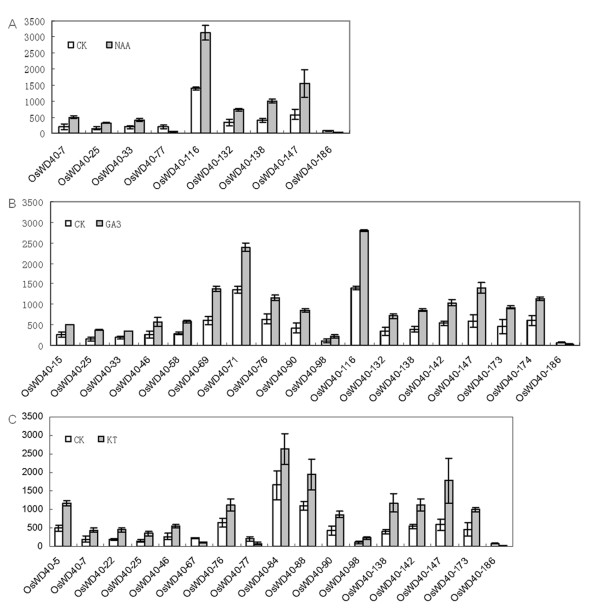
**Differential expression detected for *OsWD40 *genes in trefoil stage of seedlings showing response to treatments of three phytohormones (NAA, GA3 and KT)**. The CK indicates the expression level of corresponding *OsWD40 *genes in rice seedlings in trefoil stage without treatment. The scores are the average expression values obtained from microarrays. Error bars represent standard errors for data obtained in two biological replicates.

Phytohormones play a critical role in plant growth and development. To investigate the *OsWD40 *genes in response to phytohormone treatment, microarray analysis was performed. We identified a total of 25 *OsWD40 *genes that were differentially expressed with treatments of one or more of the phytohormone NAA, KT, GA3 in seedlings. The control indicated the expression level of corresponding *OsWD40 *genes in rice seedlings in trefoil stage without treatment (Figure 4). The fold change values with respect to control are given in Additional file 5: Table S5.

Four *OsWD40 *genes showed differential expression under all three phytohormone treatments, among which three genes (*OsWD40-25*, *138*, and *147*) were up-regulated, whereas *OsWD40-186 *was down-regulated. The expression profile of the remaining genes in response to NAA, KT, and GA3 was different. For instance, three genes (*OsWD40-33*, *116 *and *132*) were up-regulated under NAA and GA3 treatments, two genes (*OsWD40-7 *and *77*) were differentially expressed under NAA and KT treatments, and six genes (*OsWD40-46*, *76*, *90*, *98*, *142 *and *173*) were up-regulated to KT and GA3 treatment, respectively. Meanwhile, ten *OsWD40 *genes showed differential expression specifically to one phytohormone treatment. Amongst the ten genes, *OsWD40-15*, *58*, *69*, *71 *and *174 *were up-regulated specifically to GA3 treatment. We also found that *OsWD40-5*, *22*, *84 *and *88 *were up-regulated, whereas *OsWD40-67 *was down-regulated specifically to KT treatment.

The induction of *OsWD40 *genes by phytohormones prompted us to check their promoter sequence (2 kb upstream the transcript start site) by searching against the PLACE database http://www.dna.affrc.go.jp/PLACE/signalscan.html. The results suggested that all promoter regions of these 25 *OsWD40 *genes contained various elements of auxin, gibberellin, and cytokinin (Additional file 6: Table S6).

### Chromosomal localization and gene duplication

**Figure 5:**
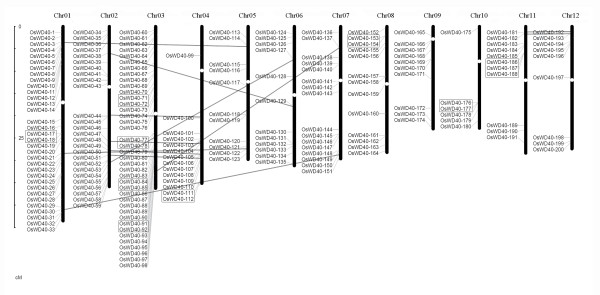
**Genomic distributions of *OsWD40 *genes on rice chromosomes**. Only gene identity numbers are provided. Grey ovals on the chromosomes (vertical bars) indicate the position of centromeres. The chromosome numbers are indicated at the top of each bar. The *OsWD40 *genes present on duplicated chromosomal segments are connected by lines and tandem duplicated genes are marked by grey shaded boxes.

The genomic distribution of *OsWD40 *genes were determined by their chromosomal positions on rice chromosome pseudomolecules. Totally, all 200 *OsWD40 *genes were dispersed on the 12 chromosomes, presenting unevenly in all regions of the chromosomes. A diagrammatic representation of chromosomal distribution of *OsWD40 *genes was depicted in Figure 5 (the exact position on rice chromosome pseudomolecules was given in Additional file 1: Table S1). Certain chromosomes had a relatively high density of *OsWD40 *genes, e.g., a maximum of 39 genes were present on chromosome 3, followed by 33 genes on chromosome 1. On the other hand, only six *OsWD40 *genes were present on chromosome 10.

Segmental duplication and tandem duplication play important roles in generating the members of a gene family during the evolution [19]. Therefore, both segmental and tandem duplication events were investigated for elucidating the potential mechanism of evolution of *OsWD40 *gene family. Analysis of the MSU RGAP rice segmental duplication database revealed only 24 (12 pairs) *OsWD40 *genes could be assigned to MSU RGAP segmental duplication blocks at a maximal length distance permitted between collinear gene pairs of 500 kb. The overall similarity of the cDNA sequences of these genes ranged from 32.7% to 96.7% and all of them were found to have their counterparts on duplicated segments (Figure 5, Additional file 7: Table S7). Nineteen *OsWD40 *genes (nine groups) seemed to be produced from tandem duplications according to the criterion adopted in our analysis (Additional file 8: Table S8). They were separated by a maximum of five intervening genes. Three group of the gene pairs were placed juxtaposed with no intervening gene. The distance between these genes ranged from 3 kb to 35 kb (Additional file 8: Table S8). Interestingly, not all the tandemly duplicated genes in the same cluster had the same direction of transcription. This might suggest the complex behavior of tandem duplications in this family. All these results suggested that much of the diversity of the *OsWD40 *gene family in rice is due to both tandem duplication and segmental genome duplication events.

**Figure 6:**
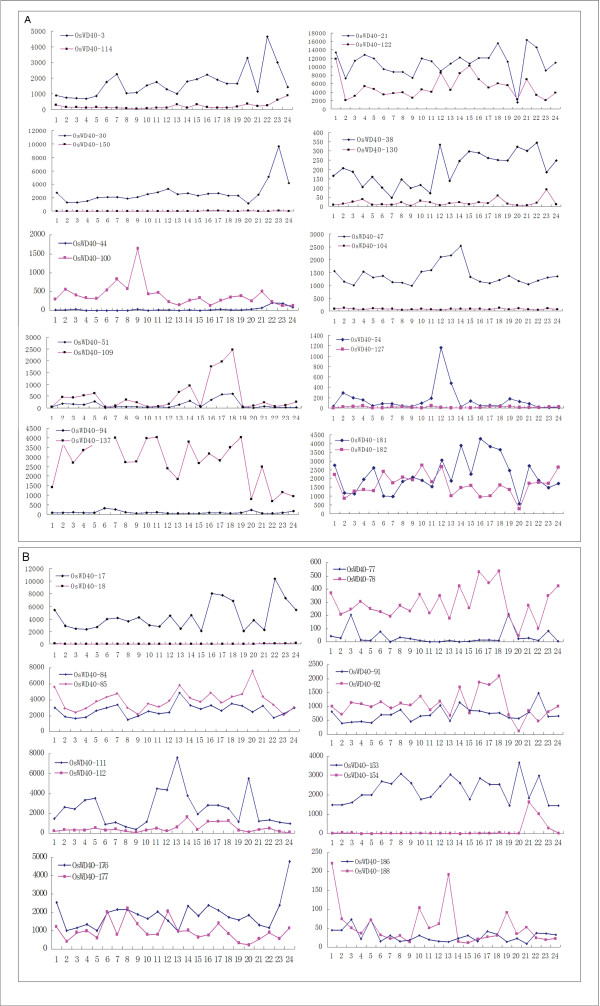
**Expression patterns of *OsWD40 *genes found in segmentally duplicated regions of the rice genome (A) and present as tandem duplicates (B)**. X-axis represents the developmental stages as given in the following table. Y-axis represents the raw expression values obtained from microarray. The detailed information of the samples is listed in Additional files 4: Table S4.

The expression patterns of *OsWD40 *genes for segmentally duplicated and tandemly duplicated genes were examined by Affymetrix rice microarray data. Probe sets were available for 19 out of 24 segmental duplication genes and 16 out of 19 tandemly duplicated genes in microarray data. A comparison of expression level revealed that two segmental duplicated genes in one pair always showed similar expression pattern, although one of the duplicated genes showed low expression level, or was not expressed at significant levels in most of the tissues (Figure 6A). We could therefore infer that immediately after segmental duplication, the two copies of genes might be functionally redundant. However, it is possible that only one of the gene copies retains its function while the other one degenerates into a pseudogene [20,21].

Two tandemly duplicated genes in Group 2 shared the same probe and *OsWD40-187 *in Group 9 did not have probe sets on Affymetrix microarray. Therefore, we analyzed the rest 16 tandemly duplicated genes in 9 groups. The expression pattern for tandem duplicated genes was more complicated (Figure 6B). The expression pattern was quite similar for four pairs of genes (Group 4, 5, 6, 8). Therefore, the gene copies might have maintained their functions during evolution, as evidenced by the similar expression pattern. Four pairs of genes (Group 1, 3, 7, 9) showed divergent expression profiles in most of the investigated tissues, as one of the genes was not expressed at significant levels in most of the tissues. This indicated that one of the members changed its function during the course of evolution. Group 7 containing *OsWD40-153 *and *OsWD40-15*4 have already been elucidated. As reported by Luo et al. [22], *OsFIE1 *(*OsWD40-154*) and *OsFIE2 *(*OsWD40-153*) were likely to have duplicated in the ancestor of the grasses. *OsFIE1 *was expressed only in endosperm with imprinted effect [23], while *OsFIE2 *was not imprinted in endosperm and was expressed constitutively. *OsWD40-18 *in Group 1 was not expressed at significant levels in all tissues, which might be induced by pseudo-functionalization after duplication [20,21].

### Expression correlation and gene ontology (GO) analyses

**Figure 7:**
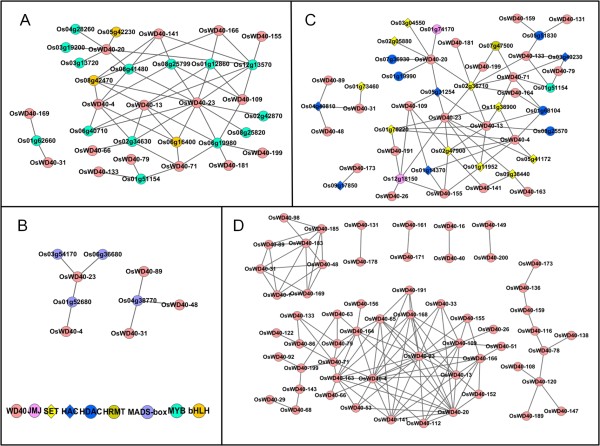
**Co-expression network composed of OsWD40 proteins and correlated genes**. (A) MYB and bHLH related genes. (B) MADS-box genes. (C) Histone-related genes. (D) *OsWD40 *genes. SET:Su (var) 3-9-Enhancer-of-zeste-Trithorax domain gene. HAC:Histone acetyltransferase. MADS-box:MADS-box transcription factor. MYB:MYB family gene. bHLH:Basic helix-loop-helix transcription factor.HDAC:Histone deacetylase. HRMT:Histone-arginine methyltransferase.

**Figure 8:**
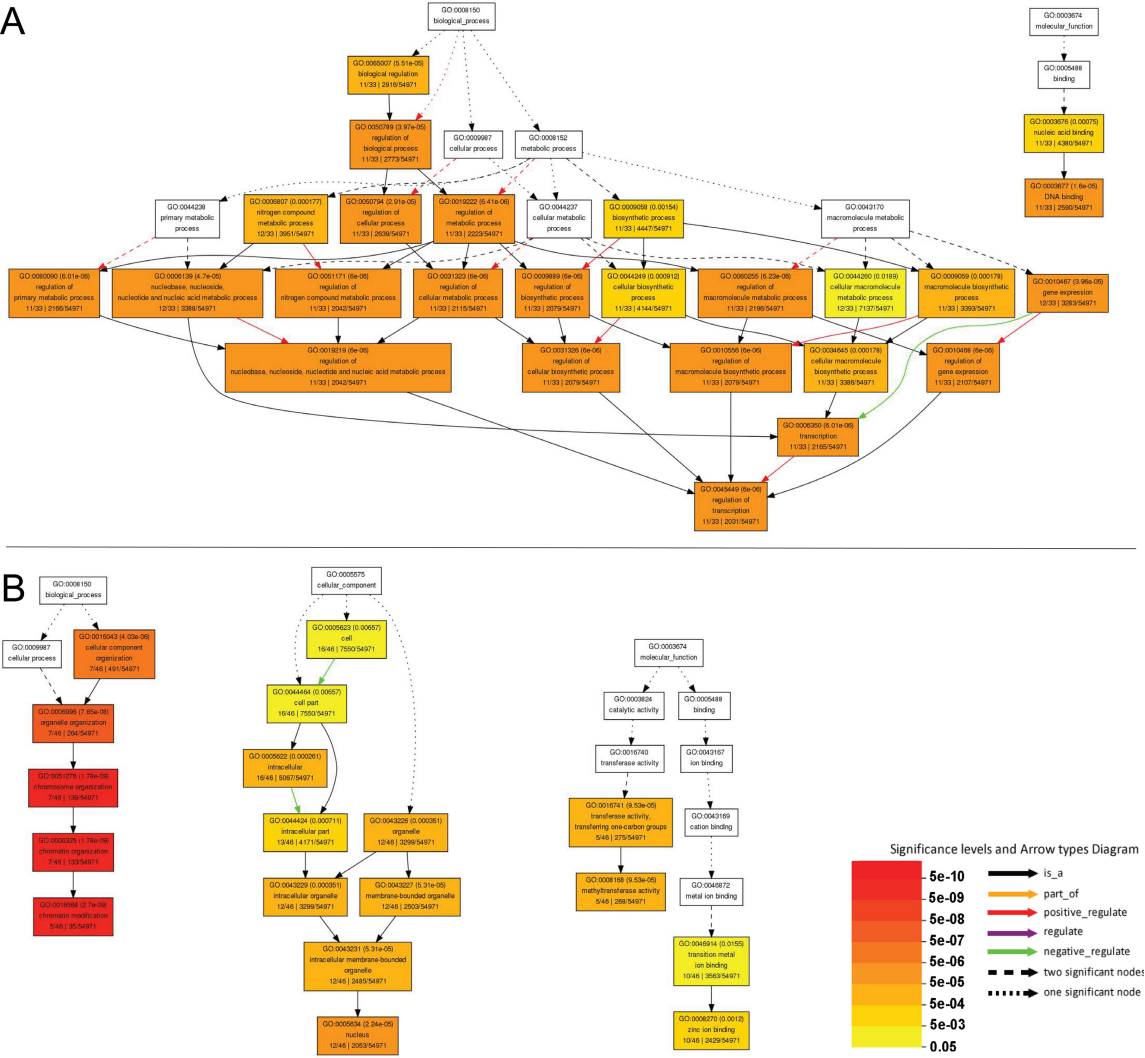
**Significant GO annotations for genes indicated in the co-expression network in Figure 7A (A) and C (B)**. The boxes in the graph list the GO identifier, the statistical significance, and the description of the GO term. The color of the box indicates the significance of the term (P < 0.05).

By using a permutation test, we set the threshold of the Pearson's correlation coefficients (PCCs) at 0.8 and extracted 2594 genes whose expression tightly correlates with 93 of the members in *OsWD40 *family (Additional file 9: Table S9). Using the functional annotation and web-based GO analysis, four networks were constructed regarding correlations between *OsWD40 *genes and their co-expressed genes, reflecting functional bias and possible molecular pathways that they involved in (Figures 7, 8, Additional file 10: Table S10).

The network in Figure 7A contained 33 rice genes (nodes) and 54 co-expression links, including 16 *OsWD40 *genes, 14 MYB related genes and three basic helix-loop-helix (bHLH) genes. Compared with the whole rice genome annotation, these *OsWD40 *genes seem to affect the regulation of multiple biological processes, such as cellular biosynthetic/metabolic process, transcription, nucleobase, nucleoside, nucleotide and nucleic acid metabolic process, gene expression, biosynthetic process, nitrogen compound metabolic process, macromolecule biosynthetic process and so on. Besides, the molecular functions related to DNA binding and nucleic acid binding were significantly enriched (Figure 8A). The network in Figure 7B contained only 9 rice genes (nodes) and 7 co-expression links. This network suggested the possibility that five *OsWD40 *genes might play roles in different molecular pathways with the participation of four MADS-box genes. Complex network has also been constructed in Figure 7C, containing 46 rice genes (nodes) and 69 co-expression links. The co-expression genes in this group were associated with histone-related proteins such as histone-lysine N-methyltransferase, histone deacetylase, single myb histone, jmjC domain containing proteins and so on. Analysis of their GO terms identified functional modules enriched for chromosome organization, chromatin organization, organelle organization, and chromatin modification. These genes also acted as important cellular components in nucleus and membrane-bounded organelle, and might take part in methyltransferase and transferase activities (Figure 8B). Our analysis also indicated that the expression of some *OsWD40 *genes was co-expressed with that of other *OsWD40 *genes. We found that 58 *OsWD40 *genes might therefore correlate with or interact with each other, thus forming a more complex feedback network (Figure 7D). However, no significant GO terms were identified.

## Discussion

### *OsWD40 *evolution and classification

Gene duplications are one of the primary driving forces during the evolution, and the variations in family size and distribution of a gene family were related to either tandem or segmental duplications [19,21]. As stated previously, *OsWD40 *family expanded from both tandem and segmental duplications, and the number of *OsWD40 *genes arranged in segmental duplications contributes to the 12% birth of new genes, while tandem duplication events contribute to the 9.5% birth of new genes.

It was interesting that different subfamilies of *OsWD40 *genes expanded in distinct manners: all segmental duplicated genes were favor for subfamily A, except *OsWD40-49 *that belonged to subfamily I. However, tandem duplications were belonged to various subfamilies, including subfamily A, B, D, G, and K.

We also noticed that the expression level of three tandemly duplicated genes, *OsWD40-77*, *84*, and *186 *were lower than that of their corresponding copies, suggesting they might lose their functions during the evolution. However, these three genes were differentially expressed under phytohormone treatments. Whereas another copy of these tandemly duplicated genes did not show differential expression under phytohormone treatments. Therefore, one would tentatively suppose that while *OsWD40-77*, *84*, and *186 *slowly lost their ancestral functions, they might evolve new functions in phytohormone pathways during the evolution.

### *OsWD40 *genes may initiate their diverse functions by performing protein complex with MYB and bHLH transcription factors

The multiple metabolism pathways in plants must be regulated by coordinated expression of different genes. The co-expression networks reflect the correlation of the expression pattern of different genes, and are suggestive in tracing the genes in the same pathway. Here, our co-expression analysis has revealed that the function of OsWD40 proteins might require the participation of various members of MYB and bHLH transcription factors (Figure 7A).

*OsWD40-13 *was found to be co-expressed with five MYB factors, Os12g13570, Os06g19980, Os01g12860, Os02g34630 and Os06g40710, as well as two bHLH factors Os06g16400 and Os08g42470. *OsWD40-13 *was homologous to plant SMU genes, which appeared to be involved in splicing of specific pre-mRNAs that affected multiple aspects of development [24]. Thus, whether transcription factors could serve as a link to mRNA process was a suggestive direction in further study. We also noticed that *OsWD40-23 *was found to be co-expressed with these transcription factors. Compared with the co-expression genes of *OsWD40-13*, four MYB factors Os02g42870, Os08g25820, Os08g25799, and Os08g41480 might be the potential interaction factors of *OsWD40-23 *in addition. *OsWD40-71 *might be a homolog of *Arabidopsis LIS*, which restricted gametic cell fate in female gametophyte [25,26]. *OsWD40-71 *was found to be co-expressed with three MYB factors, Os02g34630, Os01g51154 and Os06g19980, as well as a bHLH factor Os06g16400. These three *OsWD40 *genes were co-expressed with the same group of MYB and bHLH transcription factors, suggesting that they may take part in correlated molecular pathways by interaction with these partners.

Previous genetic analyses found that ectopic expression of maize WD40 protein PAC1 in an *Arabidopsis ttg1 *mutant was able to complement the mutant phenotypes [27], suggesting that WD40 proteins can interact with similar partners. We might speculate that some *OsWD40 *genes might be in relation with each other by controlling the expression of these transcription factors. Another *OsWD40 *gene that attracted our attention was *OsWD40-20*, which was co-expressed with another group of MYB and bHLH transcription factors that were different from the co-expression genes mentioned above. We also found that *OsWD40-20 *might be related with other partners such as genes in SET family, HDAC family, and HAC (Figure 7C). This result suggested that *OsWD40-20 *might participate in the regulation of another pathway.

A general WD/Myb/bHLH complex for regulation of the anthocyanin biosynthetic pathway was also found in *Antirrhinum majus*, *Petunia hybrida *and *Arabidopsis thaliana *[28-34]. Meanwhile, a bHLH protein Lc in maize was found to interact with MYB transcription factors to activate anthocyanin expression [35]. Another Lc-like bHLH protein was also found to require a MYB protein to perform its function [31,34,36,37]. Results suggested that many MYBs interacted directly with Lc-like bHLH proteins and the WD40 repeat protein [34,36]. Therefore, it seems that WD40 proteins allow protein-protein interactions between the bHLH and MYB proteins, and WD40 proteins in rice might also require MYBs and bHLHs to form a transcription complex to participate a range of pathways.

### *OsWD40 *genes may be involved in histone-related functions with members in SET family

Histone expression and histone post-translational modifications play pivotal roles in chromatin remodeling and epigenetic regulation in plant development [38-40]. Our co-expression analysis has revealed that *OsWD40 *genes may function with histone-related proteins. Although the exact pathways mediate by these genes are still unclear, one might speculate that these *OsWD40 *genes play important roles in histone modification.

An important group of enzymes involved in histone modification is the histone-lysine N-methyltransferases. These proteins participate in the establishment and/or maintenance of euchromatic or heterochromatic states of active or transcriptionally repressed sequences [41]. Here, a total of 10 histone-lysine N-methyltransferases were identified to be co-expressed with the OsWD40 proteins, both of which contain the SET domain that is responsible for the catalytic activity of the enzymes, suggesting possible interactions between the *OsWD40 *and SET genes in a family level. One might also tentatively speculate that the WD40 and SET domain must be the key functional structure for interaction by a conserved mechanism.

Functional studies of several *Arabidopsis *genes encoding WD40 proteins also suggest that they might be implicated in histone modification in different pathways. A WD40 domain cyclophilin, CYCLOPHILIN71 (CYP71), which functions in gene repression and organogenesis in *Arabidopsis*, serves as a highly conserved histone remodeling factor involved in chromatin-based gene silencing [42]. Another WD40 protein MSI1 in *Arabidopsis *has also been proposed to exhibit pleiotropic phenotypes by epigenetic regulation [43-45]. Therefore, *OsWD40 *genes in rice might also involved in similar pathways by histone modulation. In a word, characterization of OsWD40 proteins function in histone modification could therefore open new perspectives for understanding the molecular mechanism of epigenetic regulation.

### *OsWD40 *genes may take part in reproductive pathways with MADS-box transcription factors

*WD40 *genes identified in different plant species are involved in various developmental processes [5]. In our study, the expression patterns of *OsWD40 *genes and the co-expression analysis provide useful information for establishing their putative functions. The available evidence suggests that *OsWD40 *genes may take part in reproductive pathways with MADS-box transcription factors in rice.

MADS-box transcription factors are essential for various aspects of pathways in flower development both in dicotyledon and monocotyledon [46]. Our result suggested that *OsWD40-23 *was co-expressed with three MADS-box transcription factors, Os03g54170, Os06g36680, and Os01g52680 (Figure 7B). *OsWD40-23 *was homologous to *Arabidopsis *FVE/MSI4, a key regulator that interacted with CUL4-DDB1 and a PRC2-like complex to control epigenetic regulation of flowering time [47]. It was also reported that Os03g54170 (*OsMADS34*) was required for rice inflorescence and spikelet development [48]. Therefore, it would be interesting to investigate whether *OsWD40-23 *play roles in rice flower development with MADS-box transcription factors. We also found that three *OsWD40 *genes, *OsWD40-31*, *48*, and *89*, were co-expressed with a MADS gene Os04g38770. These four genes were expressed preferentially in stamen (Figure 2), suggesting that they may function in stamen development. All these studies support the results that MADS-box transcription factors are essential for flower developmental processes in relationship with *OsWD40 *genes.

## Conclusion

In conclusion, using an in silico approach, a total of 200 *OsWD40 *genes were found to be present in rice genome. Genomic framework revealed the potential mechanisms responsible for the evolution of *OsWD40 *genes in rice. The expression profiling of *OsWD40 *gene family covering rice life cycle could provide deep insights into their potential functions during rice growth and development. Some genes appear to be differentially expressed in different tissues/organs, vegetative and reproductive development stages, and expression of some genes is influenced under phytohormones. These data will provide the basis for understanding the evolutionary history of *OsWD40 *members and their roles in rice growth and development. The findings in our work would be useful in selecting candidate genes for functional studies of *OsWD40 *members in rice. However, future research by adopting transformation strategies or insertion mutagenesis is required to elucidate the precise functions of these *OsWD40 *genes.

## Methods

### Collection and database search of *OsWD40 *members in rice

Hidden Markov Model (HMM) profile of WD40 domain (PF00400) downloaded from Pfam http://pfam.sanger.ac.uk/ was employed to identify the putative *OsWD40 *genes in rice. The BlastP search was carried out using the HMM profile on website of MSU RGAP http://rice.plantbiology.msu.edu/, followed by removal of redundant sequences from the database. The Pfam http://www.sanger.ac.uk/Software/Pfam/ and SMART database http://smart.embl-heidelberg.de/smart/batch.pl were finally used to confirm each predicted WD40 protein. Additional conserved motifs or domains besides WD40 were identified in Pfam database. Based on these domains, we classified the OsWD40 proteins into subfamilies and the sample protein structures of each subfamily were drawn manually.

### Chromosomal localization and gene duplication

Each of the *OsWD40 *genes was mapped on rice chromosomes according to their positions available in MSU RGAP http://rice.plantbiology.msu.edu/. The distribution of *OsWD40 *genes was drawn by MapInspect http://www.plantbreeding.wur.nl/UK/software_mapinspect.html and modified manually with annotation.

The duplicated genes were elucidated from the segmental genome duplication of rice http://rice.plantbiology.msu.edu/segmental_dup/500kb/segdup_500kb.shtml, with the maximal length distance permitted between collinear gene pairs of 500 kb. Tandem duplicates were defined as genes separated by five or fewer genes. The distance between these genes on the chromosomes was calculated and the percentage of sequence similarity between the proteins encoded by these genes was determined by MegAlign software 4.0.

### Structural analysis of the *OsWD40 *genes

Information about the gene structures, transcripts, full-length cDNA, BAC accessions for each gene and characteristics of corresponding proteins were procured from MSU RGAP and KOME http://cdna01.dna.affrc.go.jp/cDNA/.

Protein sequences of putative OsWD40 members collected from the MSU RGAP and KOME were analyzed by EXPASY PROTOPARAM tool http://www.expasy.org/tools/protparam.html. Information about the number of amino acids, molecular weight, theoretical isoelectric point (pI), amino acid composition, and instability index (instability index of > 40 was considered as unstable [49]) were obtained by this tool. The conserved domain of the OsWD40 protein in rice was determined by Pfam program.

### Phylogenetic analysis of WD40 genes in rice and *Arabidopsis*

A total of 237 putative WD40 homologues in *Arabidopsis *were extracted in van Nocker and Ludwig (2003) [5]. Among which four genes were not annotated in TAIR10, thus we use 233 AtWD40 proteins in further phylogenetic analysis.

Multiple sequence alignments were performed using Clustal X version1.83 based on the full sequence of WD40 proteins from rice and *Arabidopsis *with default parameters. An un-rooted neighbor-joining phylogenetic tree [50] was constructed by generating 1,000 random bootstrap replicates using MEGA 4.

### Genome-wide expression analysis of *OsWD40 *family

Expression profile of *OsWD40 *gene family in 24 tissues for Minghui 63 was extracted from the Affymetrix rice microarray data from CREP database in our lab http://crep.ncpgr.cn. The microarray data have been submitted into the NCBI Gene Expression Omnibus (GEO) under the accession number of GSE19024 [18]. The developmental stages and organs of the tissues were described in Additional file 4: Table S4. After normalization and variance stabilization, the average signal value of two biological replicates for each sample, except for samples 2, 3, 14, 15 and 16 (three biological replicates and two technical replicates) was used for analysis. Wherever more than one probe set was available for one gene, the higher signal value of the probe sets was used for analysis. For phytohormone treatments, seedlings at trefoil stage were treated with 0.1 mM NAA, GA3 and KT, respectively. Samples were harvested at the time points of 5, 15, 30 and 60 min after treatments. The samples under the same phytohormone treatment of different time points were mixed together.

Expression values of each gene were logarithmized and cluster analyses were performed using R with euclidean distances and hierarchical cluster method of "complete linkage". The expression patterns of *OsWD40 *genes were estimated and grouped according to the hierarchical cluster. For data analysis, expression level in each of the tissues was compared against the expression in seed using a student-*t *test. The genes that are up- or down-regulated by more than two-fold and with p values < 0.05 were considered to be differentially expressed. The average expression of more than two biological replicates for each sample was used for analysis.

### Identification of correlated genes and network construction

The permutation test was done to determine the optimal threshold of the PCC [51,52]. We computed the PCCs for all pairwise relationships between the 1000 randomly selected genes in two sets of transcriptomes (expression profiles for two varieties Minghui 63 and Zhenshan 97 in CREP database of our lab, http://crep.ncpgr.cn) comprising a total of 190 microarray experiments. We estimated a nullhypothesis pairwise correlation distribution by independently permuting the components of each gene expression value and recomputing all correlations. The distribution of the PCCs before and after independent random permutation was observed to choose the optimal thresholds.

*OsWD40 *genes with standard errors greater than 500 were used for further co-expression analysis, in order to exclude the situation that the correlation of expression level was due to the constitutive expression pattern. The correlated genes with PCCs higher than the optimal thresholds were extracted from the CREP database http://crep.ncpgr.cn[18] and considered as the putative co-expression genes. The PCCs of these candidate genes were recalculated for confirmation, and the statistical significance was further determined using a student-*t*-test.

A visualization tool of Cytoscape was used to construct the co-expression network composed of the *OsWD40 *genes and their co-expressed genes. We mapped the correlated genes to the network and identified the function of *OsWD40*-correlated genes in network clusters. GO enrichment was performed by Singular Enrichment Analysis (SEA) tool in agriGO http://bioinfo.cau.edu.cn/agriGO/index.php[53] with default parameters using the rice MSU6.1 genome annotation as the background. Statistical significance was determined using the Fisher's exact test and the Yekutieli multi-test adjustment [53].

## Competing interests

The authors declare that they have no competing interests.

## Authors' contributions

YO and JY conceived and designed the research, XH and ZL collected the data. XH and YO performed the statistical analysis and analyzed the data. YO wrote the paper. All authors read and approved the final manuscript.

## Supplementary Material

Additional file 1 **Table S1 **List of 200 *OsWD40 *genes identified in rice and their sequences and protein characteristics. Click here for file

Additional file 2 **Figure S2 **Phylogenetic analysis of WD40 proteins in rice and *Arabidopsis*. The unrooted tree was generated using ClustalX by neighbor-joining method with the alignments of the OsWD40 and AtWD40 protein sequences. The five classes are marked by different colors. Scale bar represents 0.1 amino acid substitution per site. ◇: *OsWD40 *gene from subfamily K; ◆: *OsWD40 *gene from subfamily B-J. Click here for file

Additional file 3 **Table S3 **Classification of rice and *Arabidopsis *WD40 proteins based on phylogenetic analysis. Click here for file

Additional file 4 **Table S4 **The detailed information of samples used in microarry analysis. Click here for file

Additional file 5 **Table S5 **Additional file 5a. Results of differential expression analysis using seed as reference in Minghui 63. Differential expression genes have been taken p value less than 0.05 and fold change > 2 or < 0.5. When fold change > 2, regulation is up, and when fold change < 0.5, regulation is down. Additional file 5b. Results of differential expression analysis under three phytohormone (NAA, GA3 and KT) treatments in Minghui 63. Differential expression genes have been taken p value less than 0.05 and fold change > 2 or < 0.5. When fold change > 2, regulation is up, and when fold change < 0.5, regulation is down. Click here for file

Additional file 6 **Table S6 **Identification of the *cis*-elements in the promoter of *OsWD40 *genes showing response to treatments of three phytohormones (NAA, GA3 and KT). NF indicates not found. Click here for file

Additional file 7 **Table S7 ***OsWD40 *genes localized on duplicated segments of the rice genome. Click here for file

Additional file 8 **Table S8 **Tandemly duplicated *OsWD40 *genes. Click here for file

Additional file 9 **Table S9 **Additional file 9a. The distribution of the PCCs from 1000 randomly selected genes by the permutation test. Additional file 9b. The putative co-expression genes from CREP database with the Pearson's correlation coefficients higher than 0.8. Additional file 9c. The list of co-expression genes mentioned in network construction with p value less than 0.05. Additional file 9d. All connections with the Pearson's correlation coefficients higher than 0.8 (p < 0.05). Click here for file

Additional file 10 **Table S10 **Additional file 10a. GO annotations for genes indicated in the co-expression network Figure 7A (p < 0.05), with the rice MSU6.1 genome annotation as background. FDR indicates false discovery rate. P, biological_process. C, cellular_component. F, molecular_function. Additional file 10b. GO annotations for genes indicated in the co-expression network Figure 7C (p < 0.05), with the rice MSU6.1 genome annotation as background. FDR indicates false discovery rate. P, biological_process. C, cellular_component. F, molecular_function. Click here for file
