# Supplementary material for: Genomic survey, expression profile and co-expression network analysis of OsWD40 family in rice
Source: BMC Genomics. 2012 Mar 20;13:100. doi: 10.1186/1471-2164-13-100 (PMC3329404; doi:10.1186/1471-2164-13-100)
Supplement: Additional file 2 — Figure S2 Phylogenetic analysis of WD40 proteins in rice and Arabidopsis. The unrooted tree was generated using ClustalX by neighbor-joining method with the alignments of the OsWD40 and AtWD40 protein sequences. The five classes are marked by different colors. Scale bar represents 0.1 amino acid substitution per site. ◇: OsWD40 gene from subfamily K; ◆: OsWD40 gene from subfamily B-J. [file 1471-2164-13-100-S2.PPT]

## Slide 1
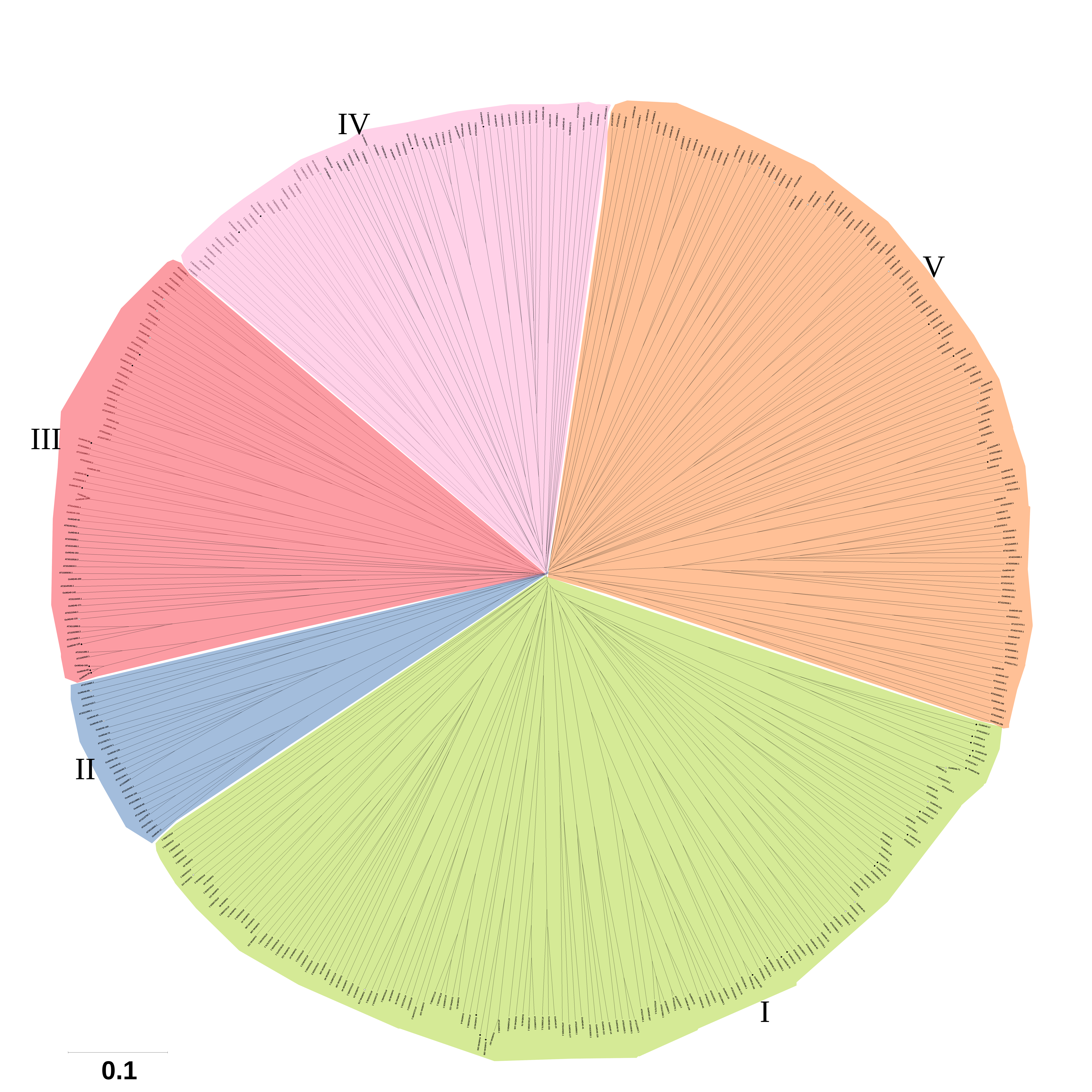

IV
AT1G21650.3
OsWD40-28
AT4G31160.1
OsWD40-135
OsWD40-15
OsWD40-180
AT4G38480.1
AT4G35140.1
AT2G46560.1
AT3G45620.2
OsWD40-63
AT3G56990.1
AT3G21540.1
AT1G19750.1
AT4G29860.1
OsWD40-67
OsWD40-56
AT1G27840.3
AT5G10940.1
OsWD40-124
OsWD40-64
AT4G34280.1
OsWD40-42
OsWD40-107
OsWD40-10
OsWD40-19
AT2G46280.1
OsWD40-172
AT2G46290.1
AT1G64350.1
OsWD40-159
OsWD40-22
OsWD40-116
AT3G49400.1
AT1G15470.1
AT3G15610.1
AT1G52730.2
OsWD40-108
AT5G16750.1
OsWD40-75
AT2G30050.1
OsWD40-130
OsWD40-38
AT3G01340.1
OsWD40-36
AT2G22040.1
AT3G18140.1
OsWD40-11
OsWD40-95
OsWD40-161
AT4G28450.1
OsWD40-139
AT2G26060.1
OsWD40-81
OsWD40-170
AT1G49540.2
AT2G30910.1
AT4G32990.1
AT5G56130.1
AT2G31300.1
OsWD40-140
AT2G01330.1
OsWD40-59
AT5G07590.1
AT3G18060.1
OsWD40-1
OsWD40-152
OsWD40-117
OsWD40-148
AT3G63460.1
AT1G18830.1
OsWD40-129
OsWD40-29
AT4G35370.1
AT4G18905.2
OsWD40-34
AT5G52250.1
AT2G32950.1
AT5G23730.1
OsWD40-55
AT2G46340.1
AT4G11110.1
OsWD40-123
OsWD40-24
AT3G15354.1
AT1G53090.1
AT3G52030.1
OsWD40-184
AT1G80710.1
OsWD40-158
OsWD40-157
AT5G58760.1
AT1G65580.1
OsWD40-2
AT2G43900.1
AT2G31830.2
AT1G05630.1
AT3G44600.1
OsWD40-164
AT3G10530.1
OsWD40-5
AT1G51690.3
AT1G17720.1
AT4G21520.1
OsWD40-88
AT1G03380.1
AT1G54710.1
OsWD40-118
AT5G54730.1
OsWD40-27
OsWD40-150
AT5G05150.1
AT3G62770.1
OsWD40-30
OsWD40-114
OsWD40-3
AT3G56440.1
AT2G40810.1
OsWD40-183
OsWD40-195
AT3G53390.1
AT2G37160.2
OsWD40-90
AT4G02660.1
AT1G03060.1
AT2G45540.2
OsWD40-105
OsWD40-48
AT1G58230.1
OsWD40-37
OsWD40-134
OsWD40-104
AT5G43930.4
OsWD40-169
AT5G64630.2
OsWD40-174
AT3G44530.1
AT5G13480.2
OsWD40-32
OsWD40-166
OsWD40-136
OsWD40-191
AT3G18860.1
AT4G29830.1
AT3G42660.1
OsWD40-181
OsWD40-192
AT3G56900.1
OsWD40-83
AT3G27640.1
OsWD40-168
AT5G05970.2
AT1G20540.1
AT1G76260.1
OsWD40-153
OsWD40-154
AT3G20740.1
OsWD40-194
AT3G05090.1
AT4G11270.1
AT1G11160.1
AT1G61210.1
OsWD40-26
AT5G08390.1
AT5G23430.1
OsWD40-111
OsWD40-179
OsWD40-138
AT2G47990.1
OsWD40-131
AT4G04940.1
OsWD40-149
AT2G34260.1
OsWD40-68
AT5G11240.1
OsWD40-197
AT2G47790.1
OsWD40-92
AT1G03110.1
OsWD40-99
AT1G08190.1
OsWD40-9
AT1G29320.1
AT4G00800.1
OsWD40-46
AT5G40880.1
AT5G49200.1
OsWD40-7
AT4G25440.1
AT5G51980.2
OsWD40-49
OsWD40-62
OsWD40-53
OsWD40-128
AT3G13290.1
AT3G13300.1
OsWD40-31
AT3G51930.1
OsWD40-77
OsWD40-198
V
III
OsWD40-40
AT5G45760.1
OsWD40-8
AT3G50590.1
AT4G31400.1
OsWD40-163
AT3G33530.3
AT2G26610.1
AT1G65030.1
OsWD40-200
AT3G49180.1
OsWD40-142
AT2G19430.1
OsWD40-171
AT5G21040.1
OsWD40-110
AT3G15980.5
AT1G52360.2
AT1G79990.1
OsWD40-125
AT2G21390.1
AT1G62020.1
OsWD40-165
OsWD40-85
OsWD40-84
AT1G19480.1
OsWD40-69
AT5G49430.1
AT2G47410.1
AT3G21060.1
OsWD40-65
OsWD40-115
OsWD40-160
OsWD40-73
AT1G78070.1
AT1G36070.1
OsWD40-126
OsWD40-162
OsWD40-52
AT5G56190.2
AT3G13340.1
AT1G55680.1
AT2G25420.1
OsWD40-155
AT3G15880.2
OsWD40-66
AT1G80490.2
AT1G15750.1
AT5G27030.2
AT3G16830.1
OsWD40-12
AT5G14050.1
AT5G05570.1
AT4G35560.2
AT3G09080.1
AT4G01860.1
OsWD40-35
AT2G40360.1
AT5G66240.2
OsWD40-146
OsWD40-143
AT5G14530.1
OsWD40-121
OsWD40-96
AT5G19920.1
AT1G10580.1
OsWD40-74
AT1G47610.1
AT2G26490.1
OsWD40-89
AT1G49450.1
AT3G18950.1
AT4G34380.1
AT3G50390.1
OsWD40-54
OsWD40-127
AT5G54520.1
OsWD40-43
OsWD40-185
OsWD40-196
AT5G02430.1
OsWD40-151
AT2G37670.1
AT5G54200.1
AT3G15470.1
OsWD40-112
OsWD40-47
AT5G42010.1
AT1G64610.1
AT5G53500.1
OsWD40-106
AT5G24320.2
OsWD40-60
AT1G48870.1
OsWD40-100
OsWD40-44
AT2G16405.1
OsWD40-25
OsWD40-80
AT4G34460.1
AT2G33340.1
OsWD40-178
AT1G18080.1
OsWD40-78
AT3G18130.1
AT1G04510.1
AT1G48630.1
AT4G21130.1
OsWD40-21
OsWD40-122
AT4G05410.1
OsWD40-120
AT1G15440.1
OsWD40-167
AT5G27945.1
OsWD40-6
OsWD40-76
AT4G29380.1
OsWD40-20
OsWD40-145
AT1G49910.1
OsWD40-156
OsWD40-41
OsWD40-57
AT1G80670.1
AT3G19590.1
AT1G69400.1
OsWD40-50
AT1G12910.1
AT1G15850.1
AT5G24520.1
AT3G26640.1
OsWD40-113
AT5G08560.1
OsWD40-14
AT5G43920.1
OsWD40-119
AT4G03020.1
OsWD40-177
OsWD40-187
OsWD40-186
OsWD40-188
AT1G24130.1
OsWD40-17
AT4G32551.2
OsWD40-4
OsWD40-18
OsWD40-58
OsWD40-103
AT2G32700.7
OsWD40-71
OsWD40-72
OsWD40-98
AT2G05720.1
OsWD40-39
AT2G41500.1
AT1G29260.1
OsWD40-132
AT5G25150.1
OsWD40-147
AT5G15550.1
OsWD40-82
AT1G71840.1
OsWD40-86
OsWD40-141
AT3G49660.1
AT5G67320.1
OsWD40-144
AT4G02730.1
OsWD40-175
OsWD40-189
AT5G52820.1
OsWD40-133
AT2G43770.1
OsWD40-45
AT5G64730.1
OsWD40-91
AT5G50230.1
OsWD40-16
AT1G49040.1
AT3G16650.1
AT4G15900.1
OsWD40-70
OsWD40-13
AT1G73720.1
OsWD40-93
AT5G60940.1
AT2G19520.1
AT4G29730.1
OsWD40-23
OsWD40-79
AT5G58230.1
OsWD40-173
AT2G16780.1
AT4G35050.1
OsWD40-182
AT2G19540.1
OsWD40-193
OsWD40-176
AT2G20330.1
OsWD40-33
AT4G11920.1
AT5G13840.1
OsWD40-51
AT4G22910.1
AT4G33260.1
OsWD40-61
OsWD40-109
AT4G33270.1
AT5G26900.1
AT5G27570.1
AT5G27080.1
AT5G50120.1
OsWD40-101
AT1G24530.1
OsWD40-102
AT5G63010.1
AT1G27470.1
AT4G07410.1
OsWD40-87
OsWD40-97
AT4G00090.1
AT3G08850.1
AT5G01770.1
OsWD40-94
OsWD40-137
AT3G52190.1
AT2G01470.1
AT5G50550.1
OsWD40-190
AT2G18900.1
AT3G26480.1
OsWD40-199
II
I
0.1
